# General and central obesity prevalence in young adult: a study based on the Rafsanjan youth cohort study

**DOI:** 10.1038/s41598-023-44579-5

**Published:** 2023-10-12

**Authors:** Mitra Abbasifard, Gholamreza Bazmandegan, Hamid Ostadebrahimi, Mahsa Amiri, Zahra Kamiab

**Affiliations:** 1 Non-Communicable Diseases Research Center, Rafsanjan University of Medical Sciences, Rafsanjan, Iran; 2 Department of Internal Medicine, Ali-Ibn Abi-Talib Hospital, School of Medicine, Rafsanjan University of Medical Sciences, Rafsanjan, Iran; 3 Department of Physiology and Pharmacology Medicine, School of Medicine, Rafsanjan University of Medical Sciences, Rafsanjan, Iran; 4 Department of Pediatrics, Ali-Ibn Abi-Talib Hospital, School of Medicine, Rafsanjan University of Medical Sciences, Rafsanjan, Iran; 5 General Physician, Rafsanjan University of Medical Sciences, Rafsanjan, Iran; 6 Department of Community Medicine, School of Medicine, Rafsanjan University of Medical Sciences, Rafsanjan, Iran

**Keywords:** Endocrinology, Risk factors

## Abstract

Growing prevalence of obesity among youth would have adverse consequences and increased risk of developing chronic diseases at older ages. This study explored the prevalence of obesity and its association with relevant risk factors in the Rafsanjan youth cohort population. This cross-sectional study was done on 3006 individuals from the 15–35-year-old population included in the Rafsanjan youth cohort study. The data were extracted from the youth cohort databases, which had been collected through in-person interview and standard questionnaires. Definition of general obesity was considered as body mass index ≤ 30 and that of central obesity as waist to hip ratio (WHR) ≥ 0.9 for men and ≥ 0.85 for women. Multivariate stepwise proportional odds model and multivariable stepwise logistic regression models were done to explore the factors associated with general obesity and central obesity. The mean age was 25.78 ± 6.06 years with 56% (n = 1683) female. The prevalence of general obesity was 15.80% (95% CI 14.50–17.11) and central obesity was 28.41% (95% CI 26.80–30.02). The risk of general obesity increased with increasing age (OR = 1.053, P < 0.0001), being married (OR = 1.658, P < 0.0001), history of diabetes (OR = 1.609, P = 0.0185), history of hypertension (OR = 1.609, P < 0.0001), elevated triglyceride (OR = 1.007, P < 0.0001) and LDL (OR = 1.015, P < 0.0001), while decreasing with being employed (OR = 0.748, P = 0.0002) and elevated HDL (OR = 0.975, P < 0.0001). Prevalence of obesity was high in study population. Marital status, increasing age, and history of chronic diseases were associated with obesity. Preventing programs should be developed against obesity and for promoting healthy habits in young adult especially during education at schools.

## Introduction

Obesity is a multifactorial metabolic disorder associated with increased adipose tissue, and is one of the most important nutritional and health problems of young adult in both developed and developing countries. Today, due to the development of urbanization, changes in lifestyle, and reduction of physical activity, the prevalence of obesity and overweight is growing^1, 2^. In addition to the negative psychological effects on obese people, research findings suggest that about 70–80% of obese adolescents change into obese adults. Obesity and overweight during young adult can increase the risk of developing chronic diseases such as hypertension, cardiovascular disease, diabetes mellitus, bone diseases, asthma, and cancer during adulthood^3, 4^. The prevalence of overweight and obesity has grown worldwide, such that one third of the world's population is now categorized as overweight or obese^2^. Obesity in children and young adult has gradually changed into a major issue in developing countries such as Iran^5^. In Iran, the prevalence of obesity during young adult is observed at high levels. The results of a review study indicate that the prevalence of obesity at ages above 18 years and below 18 years is 21.7% and 6.1%^6^, respectively. In another study, the prevalence of general obesity in Iranian children and youth was reported 11.9% overall (13.6% and 10.2% in boys and girls respectively)^7^. This trend has had a different prevalence across various provinces. In a study done in Western Azerbaijan, it was found that over recent years, the prevalence of obesity and overweight has grown in girls^8^.

Young adult ages are accompanied by rapid changes in life and behavioral patterns. These changes predispose the youth to the risk of development of high-risk health behaviors including lack of physical activity, unsuitable dietary habits, and smoking tobacco, whose effects will remain for years to come and even for the rest of life^9^. Obesity is also an important risk factor for non-communicable diseases. Considering the cumulative effects of risk factors, it has been found that the highest risk of cardiovascular disease belongs to obese cigarette smokers and especially in those with central fat accumulation^10^. Obesity during childhood and young adult increases the risk of developing different types of diseases and health issues such as diabetes mellitus, dyslipidemia, hypertension, and metabolic syndrome^11^. Obesity during childhood and young adult also causes development of adverse psychological issues such as sleep disorders, decreased self-confidence, anxiety, and depression. Research has shown that obese young adult have fewer friends compared to their peers, and spend less leisure time with their friends^12–14^.

In spite of attempts for preventing and controlling obesity, not much progress has been observed in reducing its incidence in different societies^15^. Obesity during childhood and young adult is considered to occur in response to interactions of genetic and environmental factors, risk factors such as economic factors, dietary patterns, and physical activity. Access to fatty foods, use of fast food, reduced physical activity in response to watching television and using computers, generally have varying effects on limiting or increasing the effect of the main determinant factors such as diet and physical activity in obese children^16, 17^. Considering the high and growing prevalence of obesity as well as its complications, more attention should be paid to this disease in all societies. By understanding obesity-associated factors, its occurrence and hence development of metabolic and cardiac diseases can be prevented. Thus, considering the rising trend of obesity in Iranian children and young adult, researchers should deal with the factors associated with obesity. The present study was accordingly performed to explore the prevalence of obesity and its relationship with relevant factors in Rafsanjan youth cohort population in 2021.

## Materials and methods

### Study design and sample

This cross-sectional study was done on 15–35-year-old subjects participating in youth cohort study in Rafsanjan city. The sample size of this study is 3006 individuals, chosen through cluster randomized sampling from urban and rural regions covered with four healthcare centers No. 1, 3, 4, and 8 in Rafsanjan city. The inclusion criteria for the youth cohort study included: age range 15–35 years, Iranian nationality or legal residence in Iran (having a national ID, or green card or residence card), and residence of at least 6 months of the past year in Rafsanjan. The exclusion criteria included temporary residence for occupation or education or similar cases, not understanding Persian language, and existence of severe psychological and physical disorders that would impair the course of interview. All of the participants completed written informed consent form for participation.

### Data collection

The information of this study was extracted from the databank of Rafsanjan youth cohort study, which had been collected through in-person interview and using standard questionnaires by trained experts. The results of blood analysis, anthropometric information, general information, and the medical histories of Rafsanjan youth cohort study were provided to the researcher^18^. The variables included age, gender, marital status, education, education and ethnicity of the father and mother, place of residence, occupational status, metabolic equivalent of task (MET index) (Min/week), waist circumference (WC), height, weight, body mass index (BMI), alcohol consumption over the past 12 months, cigarette smoking, and water pipe smoking. Also, histories of diseases including diabetes, hypertension, ischemic heart disease, stroke, kidney failure, liver failure, chronic obstructive pulmonary disease (COPD), thyroid disorders, and cancer were also extracted. MET index is a self-report instrument for measuring daily physical activities^19^. Based on the IPAQ recommendations for scoring protocol, participants of the study was classified in three different groups of physical activity considering the MET–min/week of the sum of walking, moderate-intensity physical activities, and vigorous-intensity physical activities: low active (< 600 MET–min/week); moderate active (≥ 600 MET–min/week) and high active (≥ 3000 MET–min/week)^20^.

### Anthropometric measurements

The weight was measured with light clothing, with no shoes, and using Seca Balance (Germany) with accuracy of 500 g. The height was also measured without shoes and via a band tape stadiometer (accuracy 1 mm). The waist circumference was measured using a non-elastic band tape with 0.1 cm accuracy on the skin, midway between the iliac crest and the lowest rib in standing position^21^. For each adolescent, the body mass index was calculated in terms of kg/m^2^; based on World Health Organization criteria, the BMI values were categorized into four groups: BMI < 18.5 underweight/thin, 18.5–24.9 normal, 25–29.9 overweight, and ≥ 30 obese^22^.

WHR was calculated as a waist to hip ratio (i.e. WC [cm]/HC [cm]). central obesity was defined as WHR ≥ 0.9 for men and ≥ 0.85 for women^21^.

The laboratory indices have been recorded using diagnostic kits of Pars Azmoon Company and BT1500 Chemistry Analyzer (Biotecnica, Italy) device in the adolescent cohort databanks.

The present study has been approved and registered with the code IR.RUMS.REC.1401.018 of in the ethics committee of Rafsanjan University of Medical Sciences.

### Statistical analysis

Results are presented as mean ± SD (standard deviation), or median (1st quartile–3rd quartile) for numeric variables, and are summarized by absolute frequencies and percentages for categorical variables. Numeric variables were compared using one-way analysis of variance (ANOVA) or independent two-sample *t* test, for normally distributed variables, or compared using non-parametric Kruskal–Wallis *H* test or Mann–Whitney *U* test, for skewed distributed variables. Categorical variables were compared using Chi-square test for trend, for ordinal variables, or Chi-square test, for nominal variables.

Multivariable stepwise proportional odds model for factors associated with BMI classification as an ordinal response including underweight, healthy weight, overweight, and obese was constructed. Also, multivariable stepwise logistic regression models for factors associated with WHR classification (normal, obese) and Waist classification (normal, central obesity) as binary responses were constructed. The associations were expressed as odds ratios (ORs) with 95% confidence intervals^23^. Variables were entered into the multivariable model if the P-value was found to be less than or equal to 0.10 in univariate analysis provided that they were logically and scientifically related to the dependent variables. Model discrimination was measured using the C statistic, which is equal to the area under the ROC (Receiver Operating Characteristic) curve. Model calibration was estimated using the Hosmer–Lemeshow (HL) goodness-of-fit statistic (higher P values imply that the model fit the observed data better).

For the statistical analysis, the statistical software SPSS version 24.0 for windows (IBM SPSS Inc., Chicago, IL, USA) and the statistical package SAS version 9.2 for windows (SAS Institute Inc., Cary, NC, USA) were used. All P-values were 2-tailed, with statistical significance defined by P ≤ 0.05.

### Ethics approval

The ethical number is IR.RUMS.REC.1400.249 which was approved by the Ethics Committee of the Rafsanjan University of Medical Sciences, Rafsanjan, Iran. Study methods were performed in accordance with the Declaration of Helsinki.

### Patient consent

Informed consent was obtained from all participants and patients’ parents included in the study.

## Results

The present study was implemented on 3006 young adult within the age range 15–35 years. Gender of more than half of the participants (56%) was female, and their mean age was 25.78 ± 6.06, and the mean of body mass index was obtained 24.80 ± 5.06 kg/m^2^. The prevalence of obesity in the Young adult based on various anthropometric indicators was shown in table 1. The prevalence of overweight and general obesity according to BMI was 28.64% and 15.80% respectively. Also, the prevalence of central obesity based on the WHR index was 28.41% (Table 1).

**Table 1:** Prevalence of obesity according to different anthropometric indicators.

Variable	n/N	Prevalence (95% CI)
BMI classification		
Underweight	287/3006	9.55 (8.50–10.60)
Healthy weight	1383/3006	46.01 (44.23–47.79)
Overweight	861/3006	28.64 (27.03–30.26)
Obese	475/3006	15.80 (14.50–17.11)
WHR classification		
Normal	2152/3006	71.59 (69.98–73.20)
Central obesity	854/3006	28.41 (26.80–30.02)

*BMI* body mass index, *WHR* waist to hip ratio, *CI* confidence interval.

The results of univariate analysis of the factors affecting general obesity based on BMI anthropometric index showed that the variables of age (P < 0.001), number of years of education (P < 0.001), father's education (P < 0.001), mother's education (P < 0.001), father's ethnicity (P = 0.040), being employed (P < 0.001), history of alcohol consumption over the past 12 months (P = 0.039), and history of cigarette smoking (P = 0.002) had a significant relationship with obesity. Also, history of having diseases including hypertension (P < 0.001), diabetes (P < 0.001), median FBS (P < 0.001), median triglyceride (P < 0.001), mean cholesterol (P < 0.001), mean HDL (P < 0.001), and mean LDL (P < 0.001) had a significant difference in four groups. The results also revealed that the odds ratio of obesity in those with moderate and high MET index was lower than that of the young adult with low physical activity (P = 0.040) (Table 2).

**Table 2:** Factors associated with obesity according to BMI anthropometric indicator in univariate analysis.

Variable	BMI classification				OR (95% CI)^#^	P-value
	Underweight (n = 287)	Healthy weight (n = 1383)	Overweight (n = 861)	Obese (n = 475)		
Age (yr)	21.38 ± 5.89	25.03 ± 5.96	27.65 ± 5.33	27.22 ± 5.89	1.097 (1.084–1.110)	< 0.001*
Gender						0.916^†^
Male (n = 1323)	124 (43.2)	618 (44.7)	376 (43.7)	205 (43.2)	Ref.	
Female (n = 1683)	163 (56.8)	765 (55.3)	485 (56.3)	270 (56.8)	1.027 (0.899–1.174)	
Years of education	11.50 ± 2.77	12.39 ± 3.05	12.63 ± 3.04	11.87 ± 2.91	1.013 (0.991–1.035)	< 0.001*
Father’s ethnicity						0.040^††^
Fars (n = 2988)	285 (99.3)	1380 (99.8)	853 (99.1)	470 (98.9)	Ref.	
Other (n = 18)	2 (0.7)	3 (0.2)	8 (0.9)	5 (1.1)	2.388 (1.025–5.565)	
Father’s years of education	8.34 ± 4.68	7.97 ± 4.88	7.45 ± 5.05	7.55 ± 4.94	0.980 (0.966–0.993)	0.014*
Mother’s ethnicity						0.512^††^
Fars (n = 2985)	285 (99.3)	1376 (99.5)	852 (99.0)	472 (99.4)	Ref.	
Other (n = 21)	2 (0.7)	7 (0.5)	9 (1.0)	3 (0.6)	1.335 (0.607–2.936)	
Mother’s years of education	7.78 ± 4.49	7.27 ± 4.71	6.78 ± 4.62	6.61 ± 4.68	0.971 (0.957–0.985)	0.001*
Marital status						< 0.001^†^
Single (n = 1328)	207 (72.1)	707 (51.1)	269 (31.2)	145 (30.5)	Ref.	
Married (n = 1630)	76 (26.5)	652 (47.1)	574 (66.7)	328 (69.1)	2.75 (2.393–3.168)	
Other (n = 48)	4 (1.4)	24 (1.7)	18 (2.1)	2 (0.4)	1.441 (0.839–2.478)	
Working (Employment)	78 (27.2)	543 (39.3)	406 (47.2)	187 (39.4)	1.318 (1.151–1.509)	< 0.001^†^
Alcohol consumption in past 12 months (n = 460)	38 (13.2)	200 (14.5)	157 (18.2)	65 (13.7)	1.117 (0.930–1.342)	0.039^†^
Cigarette smoking						0.002^†^
Never (n = 2122)	235 (81.9)	961 (69.5)	598 (69.5)	328 (69.1)	Ref.	
Former (n = 341)	16 (5.6)	158 (11.4)	107 (12.4)	60 (12.6)	1.326 (1.074–1.637)	
Current (n = 543)	36 (12.5)	264 (19.1)	156 (18.1)	87 (18.3)	1.129 (0.948–1.344)	
Hookah use in past 12 months (n = 1132)	87 (30.3)	528 (38.2)	331 (38.4)	186 (39.2)	1.130 (0.986–1.295)	0.059^†^
History of diabetes						< 0.001^†^
Yes (n = 144)	5 (1.7)	38 (2.7)	57 (6.6)	44 (9.3)	2.755 (2.024–3.750)	
No (n = 2604)	248 (86.4)	1221 (88.3)	757 (87.9)	378 (79.6)	Ref.	
I don’t know (n = 258)	34 (11.8)	124 (9.0)	47 (5.5)	53 (11.2)	0.891 (0.702–1.131)	
History of hypertension						< 0.001^†^
Yes (n = 88)	2 (0.7)	17 (1.2)	28 (3.3)	41 (8.6)	5.011 (3.361–7.470)	
No (n = 2801)	279 (97.2)	1316 (95.2)	802 (93.1)	404 (85.1)	Ref.	
I don’t know (n = 117)	6 (2.1)	50 (3.6)	31 (3.6)	30 (6.3)	1.661 (1.183–2.332)	
History of ischemic disease (n = 30)	2 (0.7)	14 (1.0)	7 (0.8)	7 (1.5)	1.265 (0.653–2.450)	0.672^††^
History of stroke (n = 18)	0	9 (0.7)	6 (0.7)	3 (0.6)	1.364 (0.583–3.190)	0.673^††^
History of renal failure (n = 26)	4 (1.4)	11 (0.8)	8 (0.9)	3 (0.6)	0.787 (0.382–1.621)	0.666^††^
History of liver failure (n = 16)	0	8 (0.6)	5 (0.6)	3 (0.6)	1.408 (0.572–3.465)	0.711^††^
History of COPD (n = 60)	5 (1.7)	35 (2.5)	12 (1.4)	8 (1.7)	0.739 (0.457–1.193)	0.267^†^
History of thyroid						0.783^†^
Yes (n = 246)	23 (8.0)	123 (8.9)	83 (9.6)	35 (7.4)	0.992 (0.784–1.254)	
No (n = 2530)	247 (86.1)	1164 (84.2)	716 (83.2)	403 (84.8)	Ref.	
I don’t know (212)	17 (5.9)	96 (6.9)	62 (7.2)	37 (7.8)	1.126 (0.870–1.459)	
History of cancer (n = 23)	0	11 (0.8)	8 (0.9)	4 (0.8)	1.450 (0.684–3.075)	0.474^††^
FBS (mg/dL)	92 (88–95)	93 (89–97)	94 (90–99)	95 (91–100)	1.033 (1.025–1.042)	< 0.001**
Triglyceride (mg/dL)	85 (66–103)	99 (77–129)	123 (94–175)	135 (103–193)	1.009 (1.008–1.010)	< 0.001**
HDL (mg/dL)	57.14 ± 9.17	56.47 ± 9.54	54.79 ± 8.85	54.00 ± 9.115	0.977 (0.970–0.984)	< 0.001*
Cholesterol (mg/dL)	149.37 ± 24.47	163.39 ± 29.74	176.10 ± 31.38	178.54 ± 28.72	1.017 (1.015–1.019)	< 0.001*
LDL (mg/dL)	74.00 ± 18.31	84.29 ± 22.70	91.96 ± 24.19	92.51 ± 22.91	1.018 (1.015–1.021)	< 0.001*
MET classification						0.040^†^
Low activity (n = 2826)	265 (92.3)	1293 (93.5)	815 (94.7)	453 (95.4)	Ref.	
Moderate to high activity (n = 180)	22 (7.7)	90 (6.5)	46 (5.3)	22 (4.6)	0.743 (0.560–0.985)	

Data are expressed as Mean ± SD (standard deviation) or Median (1st quartile-3rd quartile) or n (%).

*BMI* body mass index, *COPD* chronic obstructive pulmonary disease, *FBS* fasting blood sugar, *HDL* high-density lipoprotein, *LDL* low-density lipoprotein, *MET* metabolic equivalent, *OR* odds ratio, *CI* confidence interval.

*P-value derived from one-way ANOVA, **P-value derived from non-parametric Kruskal–Wallis *H* test, ^†^P-value derived from Chi-square test or Chi-square test for trend, ^††^P-value derived from Fisher’s exact test, ^#^OR and CI derived from univariate proportional odds model for ordinal responses.

The factors associated with general obesity based on BMI anthropometric indicator in the multivariable proportional odds model for ordinal responses in Table 3 show that the odds ratio obesity grows by 1.053 times with aging (OR = 1.053, 95% CI 1.036–1.070) (P < 0.0001). In the married young adult, the odds ratio obesity was 1.658 times that of single individuals (OR = 1.658, 95% CI 1.383–1.988) (P < 0.0001), while being lower in the employed individuals (OR = 0.748, 95% CI 0.641–0.874) (P = 0.0002). In the young adult, history of having diabetes and hypertension elevated the odds ratio of obesity by 1.069 times (OR = 1.609, 95% CI 1.157–2.239) (P = 0.0185) and 3.301 times (OR = 1.609, 95% CI 1.157–2.239) (P < 0.0001) respectively, in comparison to their counterparts without these conditions. Other results of this analysis are summarized in Table 3.

**Table 3:** Factors associated with obesity according to BMI anthropometric indicator in multivariable proportional odds model for ordinal responses.

Variable	OR (95% CI)	P-value
Age(yr)	1.053 (1.036–1.070)	< 0.0001
Marital status		< 0.0001
Single	Ref.	
Married	1.658 (1.383–1.988)	
Other	0.722 (0.406–1.283)	
Working (employment)	0.748 (0.641–0.874)	0.0002
History of diabetes		0.0185
Yes	1.609 (1.157–2.239)	
No	Ref.	
I don’t know	1.018 (0.782–1.326)	
History of hypertension		< 0.0001
Yes	3.301 (2.166–5.031)	
No	Ref.	
I don’t know	1.667 (1.146–2.424)	
Triglyceride (mg/dL)	1.007 (1.006–1.008)	< 0.0001
HDL (mg/dL)	0.972 (0.964–0.980)	< 0.0001
LDL (mg/dL)	1.015 (1.012–1.018)	< 0.0001

*BMI* body mass index, *HDL* high-density lipoprotein, *LDL* low-density lipoprotein, *OR* odds ratio, *CI* confidence interval.

Variables entered into the multivariable model including age, marital status, working (employment), cigarette smoking, alcohol consumption in past 12 months, hookah use in past 12 months, history of diabetes, history of hypertension, fasting blood sugar, triglyceride, high-density lipoprotein, low-density lipoprotein, metabolic equivalent.

The results of univariate analysis of the factors affecting central obesity based on WHR indicator showed that the variables of age (P < 0.001), gender (P < 0.001), father's education (P < 0.001), mother's education (P < 0.001), father's ethnicity (P = 0.002), marital status (P < 0.001), being employed (P < 0.001), history of alcohol consumption over the past 12 months (P < 0.001), history of cigarette smoking (P = 0.002), history of waterpipe smoking (P < 0.001) had a significant relationship. Also, history of having diseases including diabetes (P < 0.001), hypertension (P < 0.001), median FBS (P < 0.001), median triglyceride (P < 0.001), mean cholesterol (P < 0.001), mean HDL (P < 0.001), and mean LDL (P < 0.001) differed significantly between two groups. The results also indicated that the odds ratio of obesity was correlated with the number of days of doing physical activity over the past week (P < 0.001), average physical activity (min/day), and MET index (P = 0.040) (Table 4).

**Table 4:** Factors associated with central obesity according to Waist anthropometric indicator in univariate analysis.

Variable	Waist classification		OR (95% CI)^#^	P-value
	Normal (n = 2152)	Central obesity (n = 854)		
Age (yr)	24.92 ± 6.08	27.94 ± 5.42	1.093 (1.077–1.109)	< 0.001*
Gender				< 0.001^†^
Male (n = 1323)	1162 (54.0)	161 (18.9)	Ref.	
Female (n = 1683)	990 (46.0)	693 (81.1)	5.051 (4.172–6.116)	
Years of education	12.30 ± 3.07	12.29 ± 2.88	0.999 (0.974–1.026)	0.965*
Father’s ethnicity				0.002^†^
Fars (n = 2988)	2145 (99.7)	843 (98.7)	Ref.	
Other (n = 18)	7 (0.3)	11 (1.3)	3.998 (1.545–10.349)	
Father’s years of education	8.02 ± 4.93	7.21 ± 4.86	0.967 (0.951–0.983)	< 0.001*
Mother’s ethnicity				0.141^†^
Fars (n = 2985)	2140 (99.4)	845 (98.9)	Ref.	
Other (n = 21)	12 (0.6)	9 (1.1)	1.900 (0.798–4.526)	
Mother’s years of education	7.35 ± 4.71	6.38 ± 4.483	0.956 (0.940–0.973)	< 0.001*
Marital status				< 0.001^†^
Single (n = 1328)	1144 (53.2)	184 (21.5)	Ref.	
Married (n = 1630)	976 (45.4)	654 (76.6)	4.166 (3.464–5.010)	
Other (n = 48)	32 (1.5)	16 (1.9)	3.109 (1.672–5.778)	
Working (employment)	949 (44.1)	265 (31.0)	0.570 (0.482–0.675)	< 0.001^†^
Alcohol consumption in past 12 months (n = 460)	402 (18.7)	58 (6.8)	0.317 (0.238–0.423)	< 0.001^†^
Cigarette smoking				< 0.001^†^
Never (n = 2122)	1426 (66.3)	696 (81.5)	Ref.	
Former (n = 341)	276 (12.8)	65 (7.6)	0.483 (0.363–0.642)	
Current (n = 543)	450 (20.9)	93 (10.9)	0.423 (0.333–0.539)	
Hookah use in past 12 months (n = 1132)	887 (41.2)	245 (28.7)	0.574 (0.484–0.681)	< 0.001^†^
History of diabetes				< 0.001^†^
Yes (n = 144)	55 (2.6)	89 (10.4)	4.384 (3.098–6.204)	
No (n = 2604)	1902 (88.4)	702 (82.2)	Ref.	
I don’t know (n = 258)	195 (9.1)	63 (7.4)	0.875 (0.650–1.178)	
History of hypertension				< 0.001^†^
Yes (n = 88)	33 (1.5)	55 (6.4)	4.436 (2.858–6.884)	
No (n = 2801)	2036 (94.6)	765 (89.6)	Ref.	
I don’t know (n = 117)	83 (3.9)	34 (4.0)	1.090 (0.725–1.639)	
History of ischemic disease (n = 30)	19 (0.9)	11 (1.3)	1.465 (0.694–3.092)	0.314^†^
History of stroke (n = 18)	12 (0.6)	6 (0.7)	1.262 (0.472–3.373)	0.642^†^
History of renal failure (n = 26)	19 (0.9)	7 (0.8)	0.929 (0.389–2.217)	0.866^†^
History of liver failure (n = 16)	10 (0.5)	6 (0.7)	1.516 (0.549–4.183)	0.419^†^
History of COPD (n = 60)	47 (2.2)	13 (1.5)	0.692 (0.373–1.286)	0.242^†^
History of thyroid				0.470^†^
Yes (n = 246)	190 (8.8)	74 (8.7)	0.994 (0.750–1.319)	
No (n = 2530)	1818 (84.5)	712 (83.4)	Ref.	
I don’t know (212)	144 (6.7)	68 (8.0)	1.206 (0.892–1.629)	
History of cancer (n = 23)	15 (0.7)	8 (0.9)	1.347 (0.569–3.189)	0.496^†^
FBS (mg/dL)	93 (89–98)	95 (90–99)	1.021 (1.012–1.031)	< 0.001**
Triglyceride (mg/dL)	102 (80–140)	124 (95–172)	1.004 (1.003–1.005)	< 0.001**
HDL (mg/dL)	55.79 ± 9.36	55.35 ± 9.15	0.995 (0.986–1.004)	0.245*
Cholesterol (mg/dL)	164.58 ± 30.63	176.91 ± 29.83	1.013 (1.010–1.016)	< 0.001*
LDL (mg/dL)	84.38 ± 23.00	92.89 ± 23.53	1.015 (1.012–1.019)	< 0.001*
MET classification				
Low activity (n = 2826)	1995 (92.7)	831 (97.3)	Ref.	< 0.001^†^
Moderate to high activity (n = 180)	157 (7.3)	23 (2.7)	0.352 (0.225–0.549)	

Data are expressed as Mean ± SD (standard deviation) or Median (1st quartile–3rd quartile) or n (%).

*COPD* chronic obstructive pulmonary disease, *FBS* fasting blood sugar, *HDL* high-density lipoprotein, *LDL* low-density lipoprotein, *MET* metabolic equivalent, *OR* odds ratio, *CI* confidence interval.

*P-value derived from independent two-sample t test, ** P-value derived from non-parametric Mann–Whitney *U* test, ^†^P-value derived from Chi-square test, ^#^OR and CI derived from univariate logistic regression model for binary responses.

The factors associated with central obesity based on WHR index using multivariable logistic regression model for binary responses in Table 5 show that the odds ratio of central obesity grew by 1.046 times with aging. In the girls, the odds ratio of central obesity was around eight times larger than that of boys, and it was 1.925 times higher in married young adult compared to their single counterparts. In the young adult with a history of diabetes and hypertension, the odds ratio of central obesity was obtained 1.846 and 2.492 compared to subjects without these conditions respectively. The results also indicated that with increase of triglyceride and LDL, the odds ratio of central obesity would also increase, while with elevation of HDL level, the odds ratio of central obesity would decrease (Table 5).

**Table 5:** Factors associated with central obesity according to Waist anthropometric indicator in multivariable logistic regression model for binary responses.

Variable	OR (95% CI)	P-value
Age (yr)	1.046 (1.025–1.067)	< 0.0001
Gender		< 0.0001
Male	Ref.	
Female	7.984 (6.280–10.149)	
Marital status		< 0.0001
Single	Ref.	
Married	1.925 (1.505–2.461)	
Other	0.867 (0.428–1.754)	
History of diabetes		0.0005
Yes	1.846 (1.247–2.732)	
No	Ref.	
I don’t know	1.577 (1.092–2.277)	
History of hypertension		< 0.0001
Yes	2.492 (1.494–4.157)	
No	Ref.	
I don’t know	1.492 (0.905–2.461)	
Triglyceride (mg/dL)	1.006 (1.005–1.007)	< 0.0001
HDL (mg/dL)	0.969 (0.958–0.979)	< 0.0001
LDL (mg/dL)	1.016 (1.011–1.020)	< 0.0001

Hosmer–Lemeshow goodness-of-fit test; P = 0.0685.

Area under the ROC (Receiver Operating Characteristic) curve; C = 0.8036.

*HDL* high-density lipoprotein, *LDL* low-density lipoprotein, *OR* Odds ratio, *CI* confidence interval.

Variables entered into the multivariable model including age, gender, marital status, cigarette smoking, alcohol consumption in past 12 months, hookah use in past 12 months, history of diabetes, history of hypertension, fasting blood sugar, triglyceride, high-density lipoprotein, low-density lipoprotein, metabolic equivalent.

## Discussion

The growing trend of prevalence of obesity in young adult causes adverse consequences and increased risk of developing chronic conditions at older ages^4^. Obesity was previously considered a plight in high income countries. However, this trend has also gained attention as a health-threatening condition among children and young adult in developing countries as well. Unsuitable and sedentary lifestyle as well as improper nutrition results in impaired balance of energy intake and consumption, which can lead to overweight and obesity^23^.

The results of the present research showed that based on BMI and WHR, 15.8% and 28.41% of the young adult had general obesity and central obesity respectively. The results of a review study in Iran indicated that the prevalence of obesity at ages above 18 years is 21.7%, while being 6.1% at ages younger than 18^6^. In the study by Esmaeili et al., the prevalence of general obesity in Iranian children and adolescents was reported 11.9%^7^. The prevalence of obesity and overweight in a review study performed on Iranian children and adolescents was found 5.1% and 10.8% respectively^24^. Moayeri et al. in their study noted that in Tehrani adolescents based on the BMI index, the prevalence of obesity and overweight is 7.1% and 17.9% respectively^25^. In a systematic review study, the researchers stated that the prevalence of obesity in Iranian children and adolescents ranges from at least 1% to 16.1%, while the prevalence of overweight ranges from at least 4.4% up to 42.3%^26^. In the study by Janghorbani et al., based on the waist circumference index, the prevalence of central obesity in 15–34-year-old male youth was reported 3.2–7.4%, while being 18.1–44.5% in females^27^.

As the results of different studies indicated, the report of prevalence of overweight and obesity has been different based on the criterion of determining obesity and across various ages. One of these differences is related to the examined age groups. In the present study, 15–35-year-old youth have been examined, while the age range of other studies has been different. Also, since the BMI index determines the status of obesity in general, while WHR is measured for investigating central obesity, it is not unexpected to observe varying results. Another notable point is that in adolescents, due to natural increase of the waist circumference during puberty, separate cutoffs are required for determining central obesity compared to other ages, and in studies the 90 percentile of waist circumference has been proposed for them^28, 29^. Nevertheless, the high prevalence of obesity in adolescents is a major concern, since it will have adverse consequences in future^9, 10^.

The results showed that older ages, female gender, and being married correlated with increased odds ratio of obesity among young adult based on BMI and waist circumference. The results of various studies in different parts of the world have indicated that in comparison to men, women are more at risk of obesity^30, 31^. Evidence shows that men and women have differences in anatomical fat distribution, use of higher dietary fat, and obesity-associated diseases^32^. These differences can arise from genetic differences, sexual hormones, and even unknown molecular mechanisms^32, 33^. In the study by Ferreira et al., older age, low level of education, and living with a partner were among the risk factors of obesity. Physical activity of leisure time, and being accustomed to watching television more than four hours per day showed considerable effects for both genders^34^. In the study by Fonseca et al.^35^ as well as Monteiro et al.^36^, an inverse relationship was found between level of education and obesity only in women's population^37^. This association in another study suggested correlation between higher level of education and obesity as well as overweight especially in men^37^. In this regard, Kontsevaya et al. also showed that in the Russian men, there is a weak yet direct relationship between obesity and level of education, while in Russian women, higher education was associated with lower prevalence of obesity^38^.

The results of this study revealed that the adolescents who had a history of diabetes and hypertension showed higher odds ratio of general obesity and central obesity. In the study by Ferreira et al., the odds of diagnosis of hypertension, diabetes, or other non-communicable chronic diseases was higher in obese individuals, where obese men and women had a considerably higher systolic blood pressure^34^. In another study in Brazil, diagnosis of hypertension, diabetes, and angina pectoris had a positive association with obesity^39^. Considerable increase in the prevalence of obesity and diabetes among children and teenagers over the past 20 years worldwide as well as existence of a strong relationship between childhood as well as adolescent obesity and incidence of insulin resistance during early adulthood will cause accumulation of cardiovascular risk generating factors^40^. Obesity is one of the main causes of insulin resistance, and seems to have a common physio-pathological relationship with metabolic syndrome including the set of blood dyslipidemia and impaired blood glucose and blood pressure status^41^.

Based on the results of this study, elevation of triglyceride and LDL was associated with increased odds ratio of obesity among the young adult based on BMI and WHR, while elevation of HDL level was inversely correlated with odds ratio of obesity. Obese individuals are at risk of developing blood dyslipidemia and related diseases, and the children who have low body fitness and BMI have low HDL and high TG level and higher risk of developing metabolic syndrome. In the study by Korsten-Reck, around half of the children with overweight showed abnormal lipid profile including high LDL and TG as well as low HDL^42^. In the study by Bogalusa et al., it was noted that the 90 percentile of waist circumference was associated with elevated LDL cholesterol, glycaemia, insulin level, and lower HDL level^43^. The results of Kontsevaya et al. showed that there was a strong relationship between overweight as well as obesity and the indicators related to cardiovascular as well as metabolic diseases^38^. Those with obesity and overweight, regardless of age, are considerably predisposed to high blood cholesterol, low HDL, high blood triglyceride, high FBS, and high blood pressure^44^. Excessive and long-term accumulation of lipids leads to vasculitis as well as development of fatty streaks in the vessels, which gradually change into atherosclerotic plaques. Different studies have shown that higher lipid density and longer susceptibility to lipid disorders are considerably associated with the severity of atherosclerotic plaques^45, 46^. Blood dyslipidemia begins from early ages and aggravates with increase in obesity during adulthood and can predispose these people to a higher risk of atherosclerotic diseases^47^. In addition, blood dyslipidemia as one of the important components of metabolic syndrome in children, has a close association with fatty liver, nephrolithiasis, pancreatitis, and other diseases^48, 49^.

### Strength and limits

The present study has been performed cross-sectionally on a suitable sample size from the context of Rafsanjan youth cohort study. The limitations included its cross-sectional nature which leads to impossibility of examining causal relation. Accordingly, conducting longitudinal studies is suggested to explore causal relationships. Another limitation was not adjusting a series of confounding variables including diet and dietary status of the subjects since the information in this regard was not prepared. Our study may also have been exposed to recall bias since data collection has been done as self-reported.

## Conclusion

Based on the results of the study, the prevalence of obesity in young adult is considerable, since it will cause adverse consequences for the future of young adult. Accordingly, attention should be paid to this issue and the related risk factors for controlling and preventing from obesity in young adult. Preventive programs against obesity should also be developed from childhood and for promoting healthy habits in young adult.

### What is already known on this subject?

Previous studies were shown prevalence of obesity among youth would have adverse consequences and increased risk of developing chronic diseases at older ages.

### What this study adds?

The results of the present study showed that based on BMI and WHR, 15.8% and 28.41% of the young adult had general obesity and central obesity respectively. The risk of general obesity increased with increasing age, being married, history of diabetes, history of hypertension, elevated triglyceride and LDL, while decreasing with being employed and elevated HDL.

## Data Availability

The datasets in the current study are available from the corresponding author on reasonable request.

## Abbreviations

MET: Metabolic equivalent of task
COPD: Chronic obstructive pulmonary disease
SD: Standard deviation
OR: Odds ratios
ANOVA: One-way analysis of variance
HL: Hosmer–Lemeshow
BMI: Body mass index
HDL: High-density lipoprotein
LDL: Low-density lipoprotein
CI: Confidence interval
FBS: Fasting blood sugar
